# eDNA-based survey of the marine vertebrate biodiversity off the west coast of Guadeloupe (French West Indies)

**DOI:** 10.3897/BDJ.12.e125348

**Published:** 2024-06-21

**Authors:** Rachel Haderlé, Laurent Bouveret, Jordane Chazal, Justine Girardet, Samuel Iglésias, Pascal-Jean Lopez, Cédric Millon, Alice Valentini, Visotheary Ung, Jean-Luc Jung

**Affiliations:** 1 Institut de Systématique, Évolution, Biodiversité (ISYEB), Muséum national d’Histoire naturelle, CNRS, Sorbonne Université, EPHE-PSL, Université des Antilles, Paris, France Institut de Systématique, Évolution, Biodiversité (ISYEB), Muséum national d’Histoire naturelle, CNRS, Sorbonne Université, EPHE-PSL, Université des Antilles Paris France; 2 Station Marine de Dinard du Muséum National d’Histoire Naturelle, Dinard, France Station Marine de Dinard du Muséum National d’Histoire Naturelle Dinard France; 3 Observatoire des Mammifères Marins de l'Archipel Guadeloupéen (OMMAG), Port-Louis, Guadelupe (Fr) Observatoire des Mammifères Marins de l'Archipel Guadeloupéen (OMMAG) Port-Louis Guadelupe (Fr); 4 Centre international d’intelligence artificielle en acoustique naturelle, LIS, CNRS, Université de Toulon, Toulon, France Centre international d’intelligence artificielle en acoustique naturelle, LIS, CNRS, Université de Toulon Toulon France; 5 Station Marine de Concarneau du Muséum national d’Histoire naturelle, Concarneau, France Station Marine de Concarneau du Muséum national d’Histoire naturelle Concarneau France; 6 Laboratoire de Biologie des Organismes et des Ecosystèmes Aquatiques, MNHN, CNRS 8067, Sorbonne Université, IRD 207, UCN, Université des Antilles, Paris, France Laboratoire de Biologie des Organismes et des Ecosystèmes Aquatiques, MNHN, CNRS 8067, Sorbonne Université, IRD 207, UCN, Université des Antilles Paris France; 7 Spygen, Le Bourget du Lac, France Spygen Le Bourget du Lac France

**Keywords:** environmental-DNA, marine mammals, fish, West Indies, 12S mitochondrial ribosomal RNA, metabarcoding, temporal variations

## Abstract

**Background:**

In the marine environment, knowledge of biodiversity remains incomplete for many taxa, requiring assessments to understand and monitor biodiversity loss. Environmental DNA (eDNA) metabarcoding is a powerful tool for monitoring marine biodiversity, as it enables several taxa to be characterised simultaneously in a single sample. However, the data generated by environmental DNA metabarcoding are often not easily reusable. Implementing FAIR principles and standards for eDNA-derived data can facilitate data-sharing within the scientific community.

**New information:**

This study focuses on the detection of marine vertebrate biodiversity using eDNA metabarcoding on the leeward coast of Guadeloupe, a known hotspot for marine biodiversity in the French West Indies. Occurrences and DNA-derived data are shared here using DarwinCore standards combined with MIMARKS standards.

## Introduction

In the marine realm, knowledge about biodiversity is still scarce, incomplete and concerns all taxa (Mora et al. 2011, Wiens 2023). This lack of knowledge, added to the current context of biodiversity loss which impacts all ecosystems (Diaz et al. 2019) makes biodiversity assessments crucial for exploring biodiversity and understanding its erosion. Accurate analyses are needed to determine relevant conservation strategies as well as planning and monitoring this marine biodiversity (Barnosky et al. 2011). Amongst the existing strategies for implementing marine biodiversity monitoring, environmental DNA (eDNA) metabarcoding enables the simultaneous genetic characterisation of numerous taxa within a single sample using short DNA sequences (Taberlet et al. 2012, Jung 2024).

eDNA refers to DNA extracted from an environmental sample without prior isolation of organisms (Taberlet et al. 2018). Naturalistic inventories can be captured from eDNA samples using a metabarcoding approach, which assigns each eDNA molecule in the sample to its taxon (Valentini et al. 2009). eDNA metabarcoding is, thus, a powerful approach to study ecosystems that are difficult to sample and to detect rare or cryptic taxa in a non-invasive way (e.g. Bohmann et al. (2014), Ruppert et al. (2019), Günther et al. (2022)).

The records generated by eDNA metabarcoding constitute rich and complex biodiversity information. Nevertheless, most of these records are not available yet on open-science databases. Data are stored in several formats on different highly-specialised databases (or, worse, on personal computers), which confounds their re-use (Berry et al. 2021) and does not comply with the FAIR principles (Findable, Accessible, Interoperable, Reusable, Wilkinson et al. (2016)). To overcome this, the Global Biodiversity Information Facility (GBIF) has published a guide dedicated to DNA-derived occurrence data (Andersson et al. 2021), aligned with the Darwin Core framework (Wieczorek et al. 2012) and combined with the MIMARKS standards (Yilmaz et al. 2011). Using and applying these standards will enable eDNA-derived data to be shared FAIRly within the scientific community. This is particularly relevant for MOTUs' (Molecular Taxonomic Unit - a grouping of sequences, based on their molecular similarity) sequences with re-analysis and possible taxonomic re-assignment i.e. updates are crucial (Berry et al. 2021).

We have designed a study of the marine vertebrate biodiversity as reflected by eDNA metabarcoding targetted in an area of rich biodiversity, the leeward coast of Guadeloupe. The French West Indies, located in the Caribbean Sea, are a known hotspot for marine biodiversity (Bowen et al. 2013, Briggs 2007). Regarding vertebrates, more than 300 species of fish (Bouchon et al. 2002) and 21 species of cetaceans (Coché et al. 2021) have been documented in the area surrounding the Guadeloupe Archipelago. However, this area is also subject to an intense human activity, including intense maritime traffic (Madon et al. 2022), unnatural changes of the coastline (e.g. Giraud-Renard et al. (2022)) and ecotoxicological impacts (e.g.Méndez-Fernandez et al. 2018, Dromard et al. 2022, Hervé et al. 2023). However, in terms of biodiversity knowledge, the French West Indies are often considered as poorly known areas, making these areas particularly interesting to develop biomonitoring surveys.

## General description

### Purpose

The project consisted in collecting and analysing eDNA samples using, on consecutive days, the same protocol on the same transect along the west coast of Guadeloupe. Twelve samples were collected. Two sampling phases were carried out: one in 2021 over four consecutive days, the other in 2022 over two consecutive days. eDNA contained in the samples was analysed by metabarcoding using vertebrate–specific primers (Taberlet et al. 2018). The resulting dataset consisted of different lists of vertebrate taxa identified from analysed MOTUs in the different samples. Taxonomic assignments were made to the most precise taxonomic rank possible.

The project resulted in a local taxonomic inventory of marine vertebrates based on eDNA. Comparison amongst samples provided an overview of the short and middle term temporal variations in taxonomic composition at a single sampling point, as captured by our eDNA sampling and analysis protocols.

## Project description

### Funding

Data were collected during a dedicated campaign to study eDNA in the French Caribbean Archipelago of Guadeloupe, organised and financed by the UMR ISYEB and the Labex DRIIHM and benefitting from collaboration with the NGO OMMAG (Observatoire des Mammifères Marins de l’Archipel Guadeloupéen - Guadeloupe Archipelago Marine Mammal Observatory) for at-sea campaigns.

## Sampling methods

### Sampling description

Seawater samples were obtained using a protocol previously developed for freshwater samples (Taberlet et al. 2018). All samples were collected from a motorised rigid inflatable boat for 30 minutes at a 5-knots speed. For all samples, the boat followed the same transect defined on top of a marked bathymetric drop-off parallel to the coast. During each transect, two samples of seawater were collected in front of the boat, one from each side of the boat, just below the sea surface. For each sample, 30 l of sea water were continuously filtered through a VigiDNA 0.2 μm filtration capsule (SPYGEN, France) using an Athena peristaltic pump (Proactive, Hamilton, NJ, USA), as described in Dalongeville et al. (2022). Right after the completion of the procedure, each capsule was filled with 80 ml of CL1 DNA preservation buffer (SPYGEN) and stored at room temperature until DNA extraction.

### Quality control

Data were checked for errors: 10% of MOTUs were randomly selected and checked by two different persons, the taxonomic assignment was repeated and the number of reads per sample was confirmed. No errors were detected.

### Step description

DNA extraction and amplification were performed by a dedicated DNA laboratory (SPYGEN, http://www.spygen.com). PCR amplification was performed using a universal vertebrate 12S mitochondrial rDNA primer pair Vert01 (TAGAACAGGCTCCTCTAG and TTAGATACCCCACTATGC, Taberlet et al. (2018)). The amplicons were then sequenced using an Illumina MiSeq sequencer (Illumina, San Diego, CA, USA). The resulting sequence datasets (read sets) were analysed using OBITools package (Boyer et al. 2016) for taxonomic assignment.

Each MOTU was associated with a number of reads per sample. MOTUs were named using the following nomenclature: Gua_Boui_V_Year_n°MOTU; with Gua for Guadeloupe, Boui, a 4-letter code for "Bouillante" (area located on the shore the closest to the transect), V for the primer used, in this case, specific to vertebrates, the sampling year (2021 or 2022) and a number corresponding to the order of appearance of the MOTU in the overall list. The taxonomic assignment of each MOTU was meticulously checked by hand.

To compare the taxonomic resolution and the detection powers of different primers, two samples SPY210556 and SPY204197, respectively collected on the 06/06/2021 and the 06/09/2021, were also analysed with a pair of primers specific to teleosts, Tele01 (ACACCGCCCGTCACTCT, CTTCCGGTACTACCATG, Valentini et al. (2016)). Similarly, the 2021 samples (SPY204198, SPY204172, SPY210555 and SPY204197) were also analysed with a pair of mammal-specific primers, Mamm01 (CCGCCCGTCACYCTCCT, GTAYRCTTACCWTGTTACGAC, Taberlet et al. (2018)) and with a pair of cetacean-specific primers, 175f-407r (CATACGATAAGTTAAAGCTCG, GATCATTACTAGCTACCCCC, Girardet & Jung. unpublished).

## Geographic coverage

### Description

The Guadeloupe Islands are located in the Caribbean Sea, at the heart of the Agoa Sanctuary, a large marine protected area (over 143,000 km²) corresponding to the entire French Exclusive Economic Zone of the French West Indies and dedicated to the protection and conservation of marine mammals.

**Figure 1.**
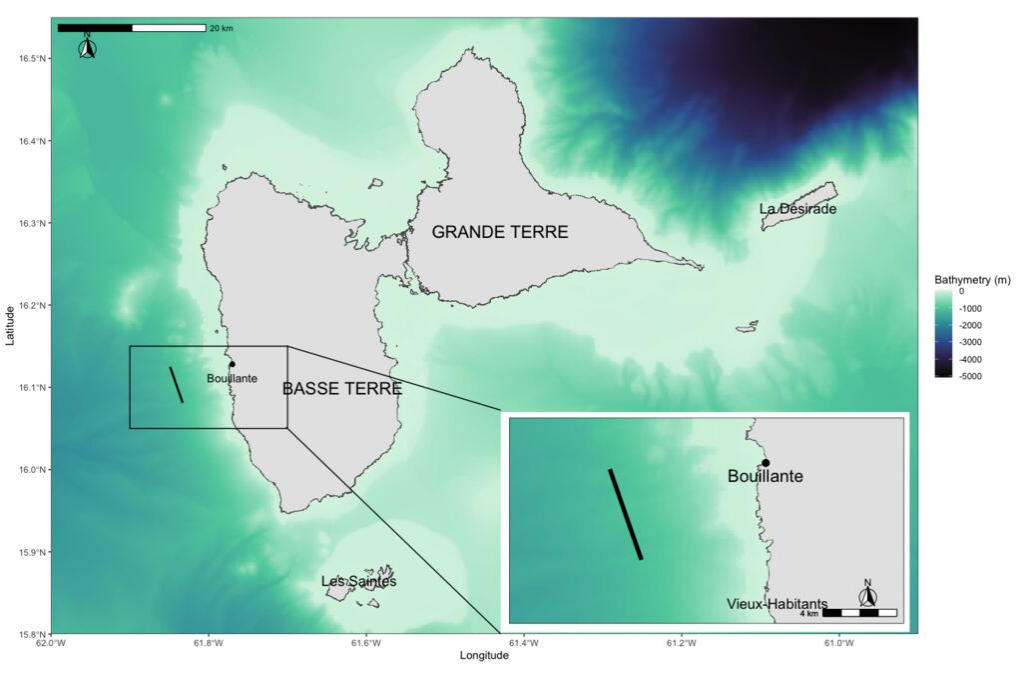
Geographical study area, the large map shows the region as a whole with bathymetry in shades of colour, the small map is a zoom showing the transect (solid black line) where the samples were collected.

The sampling area is located on the west coast of Guadeloupe Island on the Caribbean Seaside, the leeward coast, off the commune of Bouillante in Basse Terre. The sampling transect was approximately 5 km long (Fig. 1). This transect is located on a very marked bathymetric drop-off (over 1000 m deep) and links two GPS points with coordinates (16.125°, -61.849°) and (16.081°, -61.833°). This specific zone was selected because of the drop-off and numerous sightings of cetaceans, with a particular emphasis on *Physetermacrocephalus*, as regularly reported by whale watchers in this area (Coché et al. 2021).

## Taxonomic coverage

### Description

Universal primers for vertebrates were used. Some samples were also analysed using primers specific to teleosts, mammals and cetaceans. All the different taxa detected according to the primer pairs used are summarised in Table 1. All the different taxa detected according to the primer pairs used are summarised in Table 1.

## Temporal coverage

### Notes

Two sampling phases were carried out: one in 2021 on four consecutive days (from 06-06-2021 to 09-06-2021), the other in 2022 on two consecutive days (10-02-2022 and 11-02-2022).

## Usage licence

### Usage licence

Other

### IP rights notes

Data are shared under a CC-BY 4.0 licence.

## Data resources

### Data package title

eDNA marine vertebrates Guadeloupe

### Resource link

https://doi.org/10.48579/PRO/EHR5AC

### Number of data sets

2

### Data set 1.

#### Data set name

Occurrence

#### Description

This dataset contains information on each occurrence, i.e. each detection of a specific taxon in a given sample. The data includes information about the sample and the taxonomy associated with the occurrence.

**Data set 1.** 

Column label	Column description
occurrenceID	Unique identifier of the observation, named with identificationID_eventDate_eventID.
identificationID	MOTU's unique identifier (Gua_Boui_initial of the primer used_number of the MOTU).
eventDate	Sampling date (year-month-day format).
eventID	Unique identifier of the sample (SPYxxxxx).
occurrenceStatus	Statement on presence or absence, in this case "presence".
basisOfRecord	Specific nature of the data record, in this case "MaterialSample".
scientificName	Scientific name of the taxon assigned to the MOTU (this does not have to be a species, it can be any taxonomic rank) according to WoRMS taxonomy.
scientificNameID	WoRMS LSID (Life Science Identifier) of the taxon precised in scientificName.
decimalLatitude	Longitude of the midpoint of the transect in decimal degrees (EPSG:4326).
decimalLongitude	Latitude of the mid-point of the transect in decimal degrees (EPSG:4326).
footprintWKT	Transect coordinates (determined using the OBIS maptool tool).
eventRemarks	Any comments on sampling, here "port" or "starboard".
samplingEffort	Amount of effort expended during sampling, in this case "30 minutes at 5 knots".
organismQuantity	Number of reads for the MOTU in this sample.
organismQuantityType	Type of quantification system used for the MOTU, in this case "DNA sequence reads".
sampleSizeValue	Total number of reads contained in the sample.
sampleSizeUnit	Unit of measurement for the sample size, in this case "DNA sequence reads".
samplingProtocol	Description of the method used, in this case "continuous surface filtration".
identificationReferences	Reference to the bioinformatics pipeline used, in this case "OBITOOLS (Boyer et al. 2016)".
taxonRank	Taxonomic rank of the taxon assigned to the MOTU.
kingdom	Kingdom assigned.
phylum	Phylum assigned.
class	Class assigned.
order	Order assigned.
family	Family assigned (eventually).
genus	Genus assigned (eventually).
specificEpithet	Species assigned (eventually).
identificationRemarks	List of possible taxa.

### Data set 2.

#### Data set name

DNA derived data

#### Description

This dataset contains information on each occurrence, i.e. each detection of a specific taxon in a given sample. The data includes the DNA sequences associated with each occurrence as well as information on amplification, sequencing and bioinformatics analysis.

**Data set 2.** 

Column label	Column description
occurrenceID	Unique identifier of the observation, named with identificationID_eventDate_eventID.
DNA_sequence	The MOTU sequence.
target_gene	Gene where the targetted barcode is located, in this case mitochondrial "12S".
pcr_primer_forward	Sequence of the forward PCR primer used to amplify the targetted barcode sequence.
pcr_primer_reverse	Sequence of the reverse PCR primer used to amplify the targetted barcode sequence.
pcr_primer_name_forward	Name of PCR forward primer used to amplify the targetted barcode sequence.
pcr_primer_reference	Reference of PCR forward primer used to amplify the targetted barcode sequence.
env_broad_scale	Main type of environment where the sample was collected (using The Environment Ontology), in this case the "marine biome (ENVO:00000447)".
lib_layout	Nature of reads, in this case "paired".
seq_meth	Sequencing method/platform used.
otu_db	Reference database used for MOTU taxonomic assignment.

## Additional information

### Discussion and foresight

Taking into account the results obtained with vertebrates-specific primer pairs and homogenising the data from 2021 and 2022, a total of 77 different MOTUs were detected. Amongst them, 66 were identified as actinopterygians, nine as mammals and two as birds. No eDNA corresponding to another class of vertebrate was detected, including elasmobranchs. On the basis of the species lists obtained, no new taxa were identified in the geographical area. However, this conclusion must be qualified because not all MOTUs were assigned to species level, which may be explained by interspecific similarities or pre-existing gaps in the reference databases. More than 300 species of fishes have already been recorded on Guadeloupe's coasts (Bouchon-Navaro 1997) and amongst them, i.e. about 190 species, have been identifed to be associated with reefs (Bouchon-Navaro 1997). This eDNA metabarcoding inventory in Guadeloupe has, therefore, detected between one-fifth to one-quarter of the known fish diversity in this geographical area.

In order to refine the detection of actinopterygians, we have grouped them into different ecological categories (deep-sea fishes, pelagic fishes and reef-associated fishes) according to their habitat (information extracted from Fishbase, Froese and Pauly (2010)). As the samples were collected by pumping surface water on a transect located on a bathymetric drop-off more than 1,000 m deep, pelagic fishes were most likely to be detected. They represented in fact 36% of fishes detections.

In addition, a significant proportion (23%) of taxa corresponding to deep-sea fishes was also detected, for instance, *Diplospinusmultistriatus*, *Lampadenaluminosa* and *Coccorellaatlantica*. This is certainly due to their diurnal vertical migration. In fact, many deep-sea fishes move towards the upper water layers to feed at night and towards the deeper layers to avoid predation during the day (Sutton 2013). Similar results were obtained by Canals et al. (2021) who focused on the continental slope of the Bay of Biscay, where deep-sea fishes represented approximately 35% of the species richness of the epipelagic zone detected through eDNA metabarcoding. This confirms earlier statements that vertical migrations are likely to play an important role in DNA distribution patterns in marine environments (Andruszkiewicz Allan et al. 2021, Cote et al. 2023). In addition, similar detections have also been interpreted by Govindarajan et al. (2023) as a possible signature of the presence of larvae or eggs, which are known to occur at shallower depths than adults of deep-sea species (Sabatés and Masó 1990).

Reef-associated fishes represented the third ecological class of fish taxa detected during this study. The sampling area was located at around 4 km from the shore and above a deep drop-off and did not represent a possible habitat for reef fishes. The reef fishes taxonomic richness varied greatly from one sample to another (i.e. from 10 taxa detected the 02/10/2022 to no taxa detected the following day). It may be hypothesised that these detections corresponded to the larval or egg phases of these reef-associated species. Similar results have been obtained between Florida and Cuba by Kerr et al. (2020), who suggested that oceanographic processes may have transported the eggs of reef-associated fishes away from the spawning grounds and into deeper water.

Some samples were analysed with other primer pairs. For fishes, teleost primers (Valentini et al. 2016) detected more taxa (on average twice as many) than vertebrate primers. By comparing the results of the two primer pairs, certain hypotheses of correspondence can be made: for example, we can suppose that the Scombridae identified on 06/09/2021 with the vertebrate primers could correspond to one of the two taxa of the same family identified with the specific primers (*Auxissp*. or *Euthynnusalletteratus*). It appeared that certain taxa were only detected with one or the other of the primer pairs. Similarly, the study by Polanco Fernández et al. (2021) in Colombia showed similar results, suggesting that a multi-primer approach would be more effective in detecting the maximum diversity of a site (West et al. 2020).

For mammals, in general, more specific primers detected more taxa than more generalist vertebrate primers. Only mammal-specific primers perfomed an identification down to the species level: *Peponocephalaelectra* was detected on 06/06/2021, *Lagenodelphishosei* and *Stenellaattenuata* on 06/06/2021, 06/07/2021 and 06/09/2021. These specific detections can be compared with observation data from whale watchers operating in the study area. A priori, for *Stenellaattenuata* and *Lagenodelphishosei*, detections corresponded to sighting data (source: OMMAG). As for *Peponocephalaelectra*, this species is rarely observed in Guadeloupe: a priori, only 14 verified sightings in 10 years of outings (Coché et al. 2021). The successful detection of *Peponocephalaelectra* in this study could demonstrate the advantage of eDNA metabarcoding for detecting a rarely observed marine mammal group. Overall, the comparison of primers tends to show that vertebrate primers provide a general overview (fishes, birds and mammals were all detected in this study), suggesting that the primers used complement each other to reveal the biodiversity of the studied site.

## Figures and Tables

**Table 1.** List of the different taxa detected by the different pairs of primers, ordered by class and family.

**Vert01**		
Class	Family	Taxon
Actinopterygii	-	Scombriformes
Actinopterygii	Anoplogastridae	*Anoplogaster* sp.
Actinopterygii	Balistidae	*Canthidermismaculata*
Actinopterygii	Bathyclupeidae	*Neobathyclupeaargentea*
Actinopterygii	Belonidae	*Ablenneshians*
Actinopterygii	Belonidae	*Platybeloneargalus*
Actinopterygii	Belonidae	*Tylosurus* sp.
Actinopterygii	Bramidae	*Brama* sp.
Actinopterygii	Carangidae	Carangidae
Actinopterygii	Carangidae	*Caranx* sp.
Actinopterygii	Carangidae	*Decapteruspunctatus*
Actinopterygii	Carangidae	*Decapterustabl*
Actinopterygii	Chaetodontidae	Chaetodontidae
Actinopterygii	Chiasmodontidae	Chiasmodontidae
Actinopterygii	Clupeidae	*Harengula* sp.
Actinopterygii	Coryphaenidae	*Coryphaena* sp.
Actinopterygii	Epinephelidae	*Epinephelusguttatus*
Actinopterygii	Evermannellidae	*Coccorellaatlantica*
Actinopterygii	Exocoetidae	Exocoetidae
Actinopterygii	Exocoetidae	*Parexocoetus* sp.
Actinopterygii	Gempylidae	*Diplospinusmultistriatus*
Actinopterygii	Gempylidae	*Gempylusserpens*
Actinopterygii	Grammistidae	*Pseudogrammagregoryi*
Actinopterygii	Hemiramphidae	Hemiramphidae
Actinopterygii	Istiophoridae	Istiophoridae
Actinopterygii	Labridae	*Thalassomabifasciatum*
Actinopterygii	Lutjanidae	Lutjanidae
Actinopterygii	Lutjanidae	*Lutjanus* sp.
Actinopterygii	Monacanthidae	*Cantherhinespullus*
Actinopterygii	Monacanthidae	*Cantherhines* sp.
Actinopterygii	Mullidae	*Mulloidichthysmartinicus*
Actinopterygii	Mullidae	*Pseudupeneusmaculatus*
Actinopterygii	Myctophidae	*Bolinichthys* sp.
Actinopterygii	Myctophidae	*Ceratoscopelus* sp.
Actinopterygii	Myctophidae	*Diaphus* sp.
Actinopterygii	Myctophidae	*Lampadenaluminosa*
Actinopterygii	Myctophidae	*Lampanyctus* sp.
Actinopterygii	Myctophidae	*Myctophum* sp.
Actinopterygii	Neoscopelidae	*Neoscopelusmacrolepidotus*
Actinopterygii	Nomeidae	*Cubicepsbaxteri*
Actinopterygii	Ophidiidae	*Lepophidium* sp.
Actinopterygii	Pomacanthidae	*Centropyge* sp.
Actinopterygii	Pomacentridae	*Abudefdufsaxatilis*
Actinopterygii	Pomacentridae	*Azurinacyanea*
Actinopterygii	Pomacentridae	*Stegastespartitus*
Actinopterygii	Pomacentridae	*Stegastes* sp.
Actinopterygii	Scombridae	Scombridae
Actinopterygii	Scombridae	Scombrinae
Actinopterygii	Scopelarchidae	*Scopelarchoidesdanae*
Actinopterygii	Scorpaenidae	*Pteroisvolitans*
Actinopterygii	Sphyraenidae	*Sphyraenabarracuda*
Actinopterygii	Stomiidae	*Stomias* sp.
Actinopterygii	Stomiidae	Stomiidae
Aves	Procellariidae	*Ardenna* sp.
Aves	Sulidae	*Sula* sp.
Mammalia	Delphinidae	Delphinidae
Mammalia	Delphinidae	Delphininae
**Tele01**		
Class	Family	Taxon
Actinopterygii	Acanthuridae	*Acanthuruscoeruleus*
Actinopterygii	Bramidae	*Brama* sp.
Actinopterygii	Carangidae	*Caranxcrysos*
Actinopterygii	Carangidae	*Caranx* sp.
Actinopterygii	Carangidae	*Decapteruspunctatus*
Actinopterygii	Evermannellidae	*Coccorellaatlantica*
Actinopterygii	Exocoetidae	*Cheilopogon* sp.
Actinopterygii	Exocoetidae	Exocoetidae
Actinopterygii	Exocoetidae	*Parexocoetus* sp.
Actinopterygii	Hemiramphidae	*Euleptorhamphus* sp.
Actinopterygii	Hemiramphidae	Hemiramphidae
Actinopterygii	Hemiramphidae	*Oxyporhamphus* sp.
Actinopterygii	Istiophoridae	Istiophoridae
Actinopterygii	Labridae	*Xyrichtysmartinicensis*
Actinopterygii	Myctophidae	*Diaphus* sp.
Actinopterygii	Myctophidae	*Lampanyctus* sp.
Actinopterygii	Nomeidae	*Cubiceps* sp.
Actinopterygii	Pomacentridae	*Azurinamultilineata*
Actinopterygii	Scombridae	*Auxis* sp.
Actinopterygii	Scombridae	*Euthynnusalletteratus*
Actinopterygii	Stomiidae	*Astronesthes* sp.
**Mamm01**		
Class	Family	Taxon
Mammalia	Delphinidae	Delphinidae
Mammalia	Delphinidae	Delphininae
Mammalia	Delphinidae	*Lagenodelphishosei*
Mammalia	Delphinidae	*Peponocephalaelectra*
Mammalia	Delphinidae	*Stenellaattenuata*
**Cetacean-specific**		
Class	Family	Taxon
Mammalia	Delphinidae	Delphininae
